# Reconstruction of Ribosomal RNA Genes from Metagenomic Data

**DOI:** 10.1371/journal.pone.0039948

**Published:** 2012-06-27

**Authors:** Lu Fan, Kerensa McElroy, Torsten Thomas

**Affiliations:** School of Biotechnology and Biomolecular Sciences and Centre for Marine Bio-Innovation, University of New South Wales, Sydney, New South Wales, Australia; Universidad Miguel Hernandez, Spain

## Abstract

Direct sequencing of environmental DNA (metagenomics) has a great potential for describing the 16S rRNA gene diversity of microbial communities. However current approaches using this 16S rRNA gene information to describe community diversity suffer from low taxonomic resolution or chimera problems. Here we describe a new strategy that involves stringent assembly and data filtering to reconstruct full-length 16S rRNA genes from metagenomicpyrosequencing data. Simulations showed that reconstructed 16S rRNA genes provided a true picture of the community diversity, had minimal rates of chimera formation and gave taxonomic resolution down to genus level. The strategy was furthermore compared to PCR-based methods to determine the microbial diversity in two marine sponges. This showed that about 30% of the abundant phylotypes reconstructed from metagenomic data failed to be amplified by PCR. Our approach is readily applicable to existing metagenomic datasets and is expected to lead to the discovery of new microbial phylotypes.

## Introduction

Microorganisms are vital components of our planet's ecosystems. PCR amplification and sequencing of 16S ribosomal RNA (16S rRNA) genes directly from environmental samples has over the last two decades revealed an astonishing amount of new microbial diversity [1], [2]. However, as the ‘universal’ primers used in PCR are designed based on already known groups of organisms, a skewed picture of community composition is likely obtained, especially for environmental samples containing divergent microbial lineages [3].

Direct sequencing of total environmental DNA (metagenomics) has the potential to assess the true diversity of the environment without primer bias [4], [5]. Metagenomic sequences can be assigned to taxa using their similarity to reference genomes based on either sequence similarity [6]–[9] or genomic composition [10]–[13]. However, these types of assignments are only informative when the genomes of closely related taxa are present in the reference set. As reference genomes are only available for a limited part of the phylogenetic tree of life [14], these taxonomic predictions are generally of low resolution (e.g. phyla or order) and hence often give only an unsatisfactory description of community composition.

In contrast, several comprehensive databases exist for the 16S rRNA gene that provide detailed phylogenetic trees [15] and allow for taxonomic resolution down to the species level [16]. Shotgun metagenomic datasets obviously also contain fragmented 16S rRNA genes and these have been directly assigned to taxa through BLAST-based comparisons [4] or phylogenetic distance-based clustering [17]. However, the short and random nature of metagenomic sequences may not contain the phylogenetically most informative regions of the 16S rRNA genes, thus diminishing the efficiency of taxonomic assignments. Sequence assembly can potentially increase the length of the 16S rRNA gene sequences recovered [18], but low sequence coverage may limit assembly success for 16S rRNA genes and low-stringency assemblies may result in chimeric sequences [19], [20]. The recently released EMIRGE software uses iterative mapping of short Illumina reads against reference sequences to reconstruct 16S rRNA genes [19]. Although this approach has an explicit accuracy to single nucleotide difference, its potential to avoid chimeras is strongly dependent on the quality of the reference database. Further, EMIRGE's algorithm is currently not designed for pyrosequencing reads, which contain high rates of insertion and deletions errors (e.g. in homopolymers) [21]. There is thus a need for an approach that reconstructs 16S rRNA genes with high accuracy from pyrosequencing data.

In the present study, we describe a strategy to reconstruct nearly full-length 16S rRNA sequences from metagenomicpyrosequencing data. Through simulation of communities with different diversities we developed a process of stringent assembly and data filtering that generates 16S rRNAcontigs with minimal chimera rates. We then applied our process to assess the microbial symbiont communities from two marine sponges species and compared the outcome to PCR-based assessments of the community structure (pyro-tag-sequencing). We show that about 30% of the abundant phylotypes reconstructed from metagenomic reads failed to be amplified by PCR, which is most likely due to primer mismatches.

## Materials and Methods

### Simulated metagenomes and metagenomic samples

Ninety completed genomes were selected as references, including 76 bacteria and 14 archaea and combined using established profiles of community diversity with high- (HC), median- (MC), and low- (LC) complexity [22] (Table S1). Genomic sequences, 16S rRNA gene sequences and gene copy number per genome were obtained from the Integrated Microbial Genomes website (http://img.jgi.doe.gov/cgi-bin/w/main.cgi). Heterogenous 16S rRNA genes within a genome were considered separately. For each metagenome complexity, three read data set (1,000,000 reads each, 350 nt) were simulated using empirically derived and context-based error models (GemSIM software [23]).

Three environmental DNA samples for each of the two sponges *Cymbastelaconcentrica* and *C. coralliophila* were obtained as described in ref. [24]. Shotgun pyrosequencing (454 Titanium) was conducted at the J. Craig Venter Institute, Rockville, USA and the resulting average read length corresponded to the simulated datasets above. The shotgun sequencing is available through the Community Cyberinfrastructure for Advanced Microbial Ecology Research and Analysis website (http://camera.calit2.net/) under project accession ‘CAM_PROJ_BotanyBay’.

### Reconstruction of 16S rRNA gene sequences

The metagenomic reads of the simulated communities and the sponge microbial communities were pre-processed with PrinSeq [25] using the settings ‘(“minlen”:“60”,“maxlen”:“700”,“minqualm”:“20”,“nsmaxp”:“1”,“complval”:“50”, “noniupac”:“true”,“derep0”:“true”,“derep1”:“true”,“complmethod”:“2”,“trimtails”:“6”,“trimns”:“1”,“trimscore”:“15”,“trimwindow”:“2”,“trimstep”:“1”,“tailsite”:“1”,“trimsite”:“3”,“trimtype”:“2”,“trimrule”:“1”)’. Metaxa (version 1.0.2) [26] was then used to identify reads containing 16S rRNA sequences. Reads (>300 nt) from triplicates were then pooled and assembled with the GS De Novo Assembler 2.3 (454 Life Sciences, Branford, CT) using the ‘cDNA’ option, which is optimized for the uneven and high coverage typically expected in RNA assemblies. Default settings were used except ‘overlap identity’, which was set to 99%. Additionally, ‘reads limited to one contig’ and ‘extending low depth overlaps’ were selected. The 99% cut-off was chosen to allow overlap of reads with a 1% error, which is typical seen towards the end of pyrosequencing reads [23]. Lower stringency (e.g. 97% as used by Radax*et al.* during the assembly of 16S rRNA gene [27]) resulted in unacceptable rates of chimera formation (data not shown). After aligning contigs to the SILVA 1.08 database by SINA [28], flanking regions that were not part of the 16S rRNA gene sequences were removed. Resulting contigs were then examined for chimerism. If a contig constituted reads from more than one strain and any of these strains was less than 99% sequence identity to the other strains, it was considered a chimera.

### Pyrosequencing of 16S rRNA genes amplified by PCR

Amplification of the 16S rRNA gene was performed on the same DNA sample as used for shotgun sequencing. Primers 28F ‘GAGTTTGATCNTGGCTCAG’ and 519R ‘GTNTTACNGCGGCKGCTG’ were used for amplification of the variable regions V1-3. PCR and subsequent sequencing are described in Dowd *et al.* 2008 [29] and were performed at the Research and Testing Laboratory (Lubbock, USA). Trace data was deposited at the NCBI Sequencing Read Archive database with the project accession SRP011939.

Analysis of the 16S rRNA tag-sequencing data was performed using Mothur v1.23.1 [30]. Specifically, ‘shhh.flows’ was used for de-noising, ‘trim.seqs (pdiffs = 2, bdiffs = 1, maxhomop = 8, minlength = 200)’ was used for barcode removal and quality filtering, SINA was used for sequence alignment with the SILVA 1.08 database [28], ‘screen.seqs(start = 1048, minlength = 245)’ and ‘filter.seqs (vertical = T, trump = .)’ were used for alignment quality filtering, ‘pre.cluster(diffs = 2)’ was used for further error reduction, ‘chimera.uchime’ was used for *de novo* removal of chimeric reads, and Metaxa (version 1.0.2) [26] was used to remove mitochondrial and chloroplast sequences.

### Operational taxonomic unit (OTU) analysis

For simulated data, filtered 16S rRNAcontigs (with coverage of more than 10 reads and length greater than 700 nt; see below) and 16S rRNA reads not in contigs were pooled with the 16S rRNA sequences of the reference genomes used for simulation. Redundancy within these pools was removed with CD-hit (99% identify cut-off). PhylOTU [17] was then used to generate OTUs with 0.01, 0.03 and 0.05 phylogenetic distance cut-off. OTUs containing both reference sequences and simulated shotgun sequences (filtered contigs or reads) were assigned as ‘recovered’. OTUs containing only reference sequences were termed as ‘missed’, while those containing only shotgun sequences were assigned as ‘artificial’. OTU coverage was defined as the number of reads contained in each OTU. For the sponge samples, filtered 16S rRNAcontigs (with coverage of more than 10 reads and length greater than 700 nt) and 16S RNA reads not in contigs were pooled with PCR-amplified tag-sequences and then processed as above to generate OTUs. Diversity analysis was performed with QIIME [31] and phylogenetic distance-based rarefaction was based on the tree of non-redundant sequences generated during the PhylOTU process.

### Taxonomic classification and phylogenetic analysis

16S rRNA classification was performed with the RDP Classifier 2.3 [32], except for the classification of the abundant OTUs in sponge samples, which was performed with the Greengenes Classifier (March 6, 2012) [33] followed by manual examination. Single-copy gene based analysis was performed using MLTreeMap (version 2.05, ‘minimal sequence length after Gblocks’ set to 35) [7]. For phylogenetic analysis, Maximum-Likelihood trees of the 16S rRNA gene contigs were constructed using RAxML [34] after alignment by SINA and removal of ambiguous positions by Gblocks (−t = d −b4 = 5 −b5 = h) [35].

## Results

### 16S rRNA gene assembly with minimal chimera formation

As chimera formation was a major issue in previous assembly approaches [18], [19], [27], we first examined the occurrence of chimeric 16S rRNAcontigs in our assembly strategy on simulated datasets (see Materials and Methods). 9,931 (0.11%) reads containing 16S rRNA gene information were detected from 8,997,875 shotgun reads after quality filtering (Table 1). After applying our assembly strategy we recovered between 125–130 contigs containing full or partial 16S rRNA genes (Table 1).

**Table 1:** Reads, 16S rRNAcontigs, OTUs and chimera examination of the simulated communities.

Sample	HC–A	HC–B	HC–C	MC–A	MC–B	MC–C	LC–A	LC–B	LC–C
**Reads after quality filtering**	999913	999909	999912	999703	999775	999769	999603	999606	999685
**16S rRNA gene – containing reads**	1303	1353	1376	984	1112	1153	874	916	860
**16S rRNAcontigs> 350 nt (chimera, chimera containing >1 contaminating read)**	130 (3, 1)			126 (7, 1)			125 (4, 3)		
**Reads in 16S rRNAcontigs>350 nt (chimera, chimera containing >1 contaminating read)**	3733 (85, 15)			3005 (365, 8)			2386 (374, 150)		
**Filtered 16SrRNAcontigs (chimera, chimera containing >1 contaminating read)**	73 (0, 0)			53 (3, 0)			54 (3, 2)		
**Reads in filtered 16SrRNAcontigs (chimera, chimera containing >1 contaminating read)**	3257 (0, 0)			2610 (330, 0)			2004 (364, 140)		
**Length of filtered 16S rRNAcontigs (min, max, mean) (nt)**	458, 1548, 1262			574, 1529, 1127			515, 1532, 1174		
**Recovered, missed, artificial OTUs (0.01)**	81, 0, 0	81, 0, 0	81, 0, 0	75, 1, 1	77, 1, 1	77, 1, 1	80, 0, 0	79, 0, 0	80, 0, 0
**Reads in recovered, missed, artificial OTUs (0.01)**	1303, 0, 0	1353, 0, 0	1376, 0, 0	978, 2, 4	1106, 2, 2	1148, 4, 4	870, 0, 0	915, 0, 0	857, 0, 0
**Recovered, missed, artificial OTUs (0.03)**	74, 0, 0	74, 0, 0	74, 0, 0	69, 0, 0	72, 0, 0	72, 0, 0	72, 0, 0	71, 0, 0	72, 0, 0
**Reads in recovered, missed, artificial OTUs (0.03)**	1303, 0, 0	1353, 0, 0	1376, 0, 0	982, 0, 0	1108, 0, 0	1150, 0, 0	870, 0, 0	915, 0, 0	857, 0, 0
**Recovered, missed, artificial OTUs (0.05)**	52, 0, 0	53, 0, 0	52, 0, 0	49, 0, 0	50, 0, 0	49, 0, 0	49, 0, 0	48, 0, 0	48, 0, 0
**Reads in recovered, missed, artificial OTUs (0.05)**	1303, 0, 0	1353, 0, 0	1376, 0, 0	982, 0, 0	1108, 0, 0	1150, 0, 0	870, 0, 0	915, 0, 0	857, 0, 0

**Figure 1:**
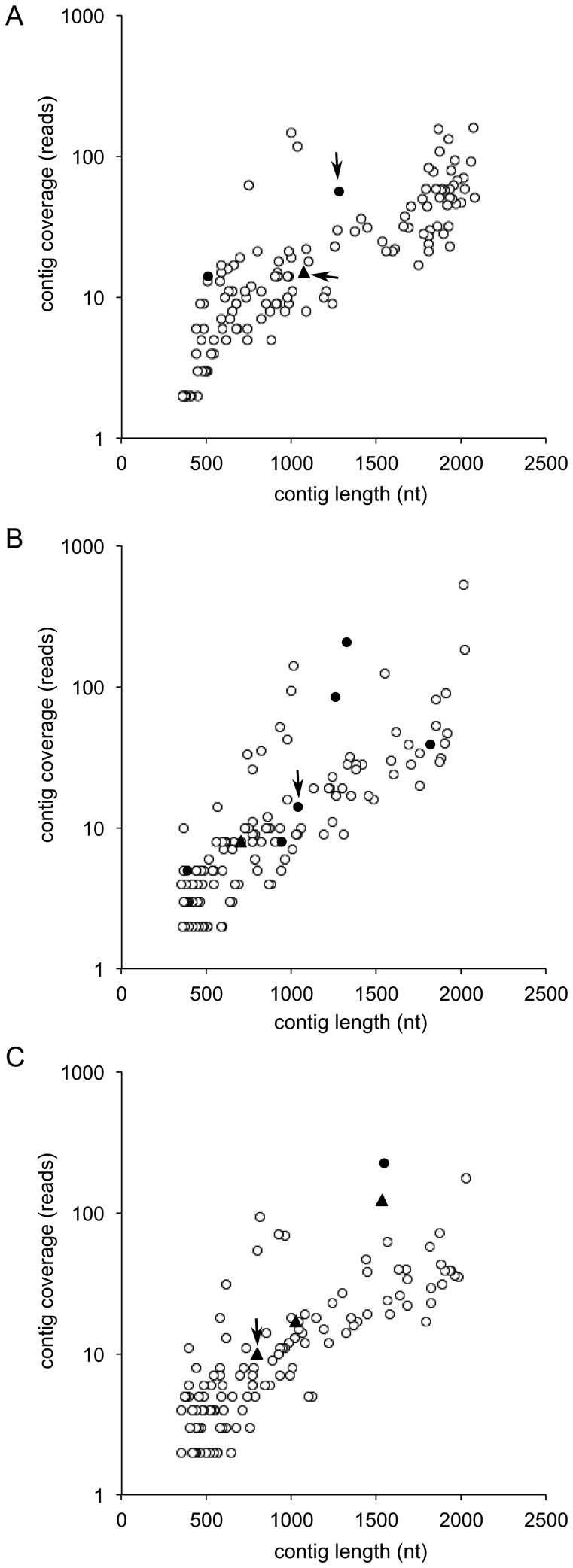
16S rRNA gene contigs and chimeric contigs for simulated datasets. Open circle: non-chimeric contigs; solid circle: chimeric contigs containing one contaminating read; solid triangles: chimeric contigs containing more than one contaminating read. Arrow: chimera detected by UChime. (A) HC. (B) MC. (C) LC.

16S rRNAcontigs larger than 350 nt were plotted by their length and read coverage (Figure 1). Fourteen chimeric contigs (3.6%) were detected in all 381 contigs generated from the nine datasets (solid circle and triangles in Figure 1). Four of these contigs could be readily detected using UChime [36] (arrows in Figure 1). Eight chimeras contain only one ‘contaminating’ read (solid circles in Figure 1), which were mostly aligned to highly conserved regions of the 16S rRNA gene (data not shown). To examine whether these chimeras would affect the accuracy of community structure prediction, we generated OTUs with different phylogenetic distance cut-offs (0.01, 0.03 and 0.05). In nearly all case, all reference OTUs were recovered and no artificial OTUs were generated. The only exception was for MC communities at a 0.01 OTU level where one artificial OTU was generated and one OTU present in the reference was missed (Table 1). This result shows that our assembly strategy recovers effectively the true microbial community structure, and especially OTU groupings of greater than 0.03 phylogenetic distance.

With the aim of recovering long 16S rRNA sequences for phylogenetic analysis and to minimize the effects of potential chimeric assembly, we filtered contigs for length of greater than 700 nt and for a coverage of more than 10 reads (Figure 1). In addition we used UChime for chimera removal. Sequences flanking the 16S rRNA gene were removed. This resulted in 180 contigs (mean length: 1,174–1,262 nt) in the nine samples with only two (1.1%) of them containing more than one contaminating read (Table 1). This value is below the chimeric amplification rate generally reported for PCR-based assessment of 16S rRNA gene diversity (5 to 45%) [5], [37]–[40].

### Assembly of 16S rRNA sequences improves taxonomic classification

**Figure 2:**
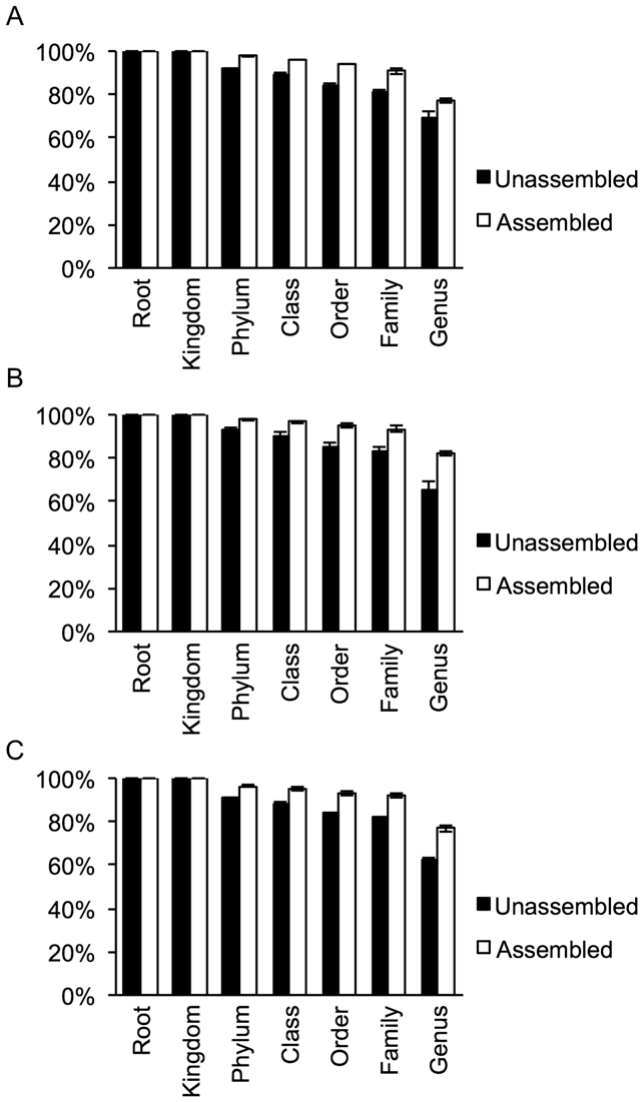
Taxonomic classification of assembled and unassembled shotgun 16S rRNA gene reads for simulated datasets. (A) HC. (B) MC. (C) LC.

With the assumption that longer 16S rRNA gene sequences can improve the taxonomic description of a community, we compared the proportion of reads before and after assembly that could be confidently assigned using the RDP Classifier (80% confidence). Despite all strains in the simulated datasets being deposited in the RDP database, a steady decline of classification success was observed with between 60–70% of unassembled reads being assigned at the genus level. In contrast, assembled data showed generally higher classification success and at genus level more than 80% could be confidently assigned (Figure 2). This shows a clear benefit of 16S rRNA gene assembly for taxonomic classification and will also improve phylogenetic analysis (see below).

### 16S rRNA gene reconstruction reveals community diversity that is missed by PCR-based approaches

Sponges (phylum Porifera) host complex communities of microbial symbionts, which are essential for the host's function [41]. Over the last decade substantial efforts have been made to describe the phylogenetic diversity and biogeography of sponge-associated microorganisms [41], [42]. However, the vast majority of sponge microbiome surveys are based on PCR-amplification of the 16S rRNA gene. Only recently has one study generated 16S rRNAcontigs from a shotgun-sequenced transcriptome of a sponge microbial community [27]. However, this study generated relatively short contigs (729 nt on average) despite extremely high sequencing coverage (66,743 reads containing 16S rRNA gene sequences) and the loose stringency during assembly could have created many chimeras (see above) [27].

To evaluate the phylogenetic diversity generated by our 16S rRNA gene reconstruction method, we analyzed six shotgun metagenomes from the two sponges*C. concentrica* and *C. coralliophila*. From 5,322,385 quality-filtered pyrosequencingreads, we could identify 1,942 reads containing 16S rRNA genes (0.04%) and generated 25 filteredcontigs (Table 2). The majority of contigs were full or near-full length (Table 2). Community composition of the six sponge DNA samples was also assessed by PCR-amplifying and pyrosequencing the variable region V1-3 of the 16S rRNA gene (pyro-tag-sequencing). 22,392 16S rRNA gene sequences were obtained and 1,366 were unique sequences after quality filtering and pre-clustering (Materials and Methods, Table 3).

**Table 2:** The sponge metagenomic datasets.

Sample	Cyr–A shotgun	Cyr–B shotgun	Cyr–C shotgun	Cyn–A shotgun	Cyn–B shotgun	Cyn–C shotgun
**Sponge host**	*C. coralliophila*			*C. concentrica*		
**Raw reads**	897408	971976	888127	678263	1169872	1323699
**Average read size (nt)**	387.6	353.2	276.8	358.0	408.1	392.8
**Reads after quality filtering**	859525	898161	788662	660869	1004075	1111093
**16S rRNA gene – containing reads**	282	385	95	237	530	413
**16S rRNA gene contigs>350 nt (reads)**	48 (557)			66 (908)		
**Filtered 16S rRNA gene contigs (reads)**	13 (445)			12 (727)		
**Length of filtered 16S rRNA gene contigs (min, max, mean) (nt)**	1218, 1535, 1418			493, 1517, 1251		

**Table 3:** The sponge tag-sequencing data sets.

Sample	Cyr–A PCR	Cyr–B PCR	Cyr–C PCR	Cyn–A PCR	Cyn–B PCR	Cyn–C PCR
**Sponge host**	*C. coralliophila*			*C. concentrica*		
**Raw reads**	5989	7895	13961	8257	5284	12509
**Average read size (nt)**	301.1	302.5	305.7	306.8	317.2	314.1
**Reads after quality filtering**	2342	3038	4988	3754	2140	6130
**Unique sequences**	212	179	311	265	155	244
**Average size of unique sequences (nt)**	269.8	268.9	272.2	267.2	271	269.2

**Figure 3:**
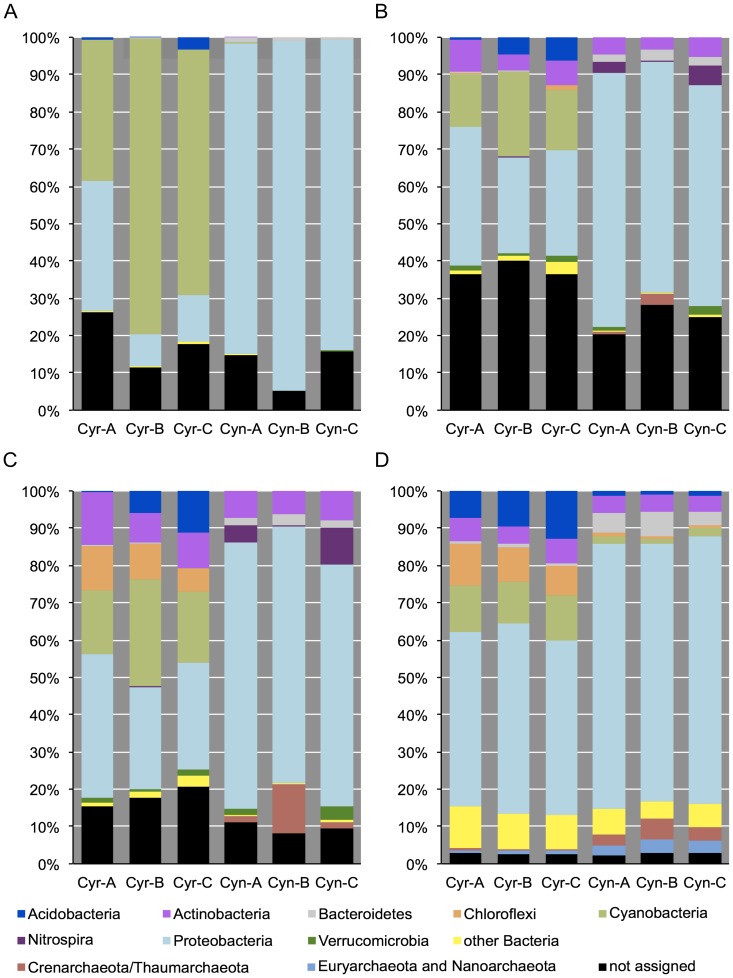
Phylum-level classification of the sponge pyro-tag-sequencing and shotgun sequencing datasets. (A) 16S rRNA gene PCR approach. (B) Unassembled shotgun 16S rRNA gene reads. (C) Assembled shotgun 16S rRNA gene reads. (D) Single-copy gene analysis.

We first compared community composition derived from the pyro-tag-sequencing data, the shotgun reads with and without assembly and single-copy genes (Material and Methods) at the phylum level (Figure 3). In general, more phyla were detected in shotgun sequencing reads compared to pyro-tag-sequencing data. Specifically, the PCR-based approach using the 28F/519R primer set recovered predominately phylotypes belonging to cyanobacteria and proteobacteria, while the shotgun data also detected sequences in Actinobacteria, Nitrospira, Chloroflexi, and Verrucomicrobia (Figure 3A, B). This may be not only due to potential primer bias (see below), but also the short sequences (∼250nt after quality processing) (Materials and Methods, Table 3) that are difficult to classify. The presence of these ‘missed’ phyla (e.g. Chloroflexi) was also confirmed by single-copy gene based search (Figure 3D). However, this single-copy gene approach also failed to detect some taxa (e.g. Nitrospira and Verrucomicrobia), which is likely due to the low number of reference genomes available for these phyla. Overall, these results show that 16S rRNA gene analysis from metagenomic datasets has superior capacity to detect a broad range of phylogenetic diversity.

**Figure 4:**
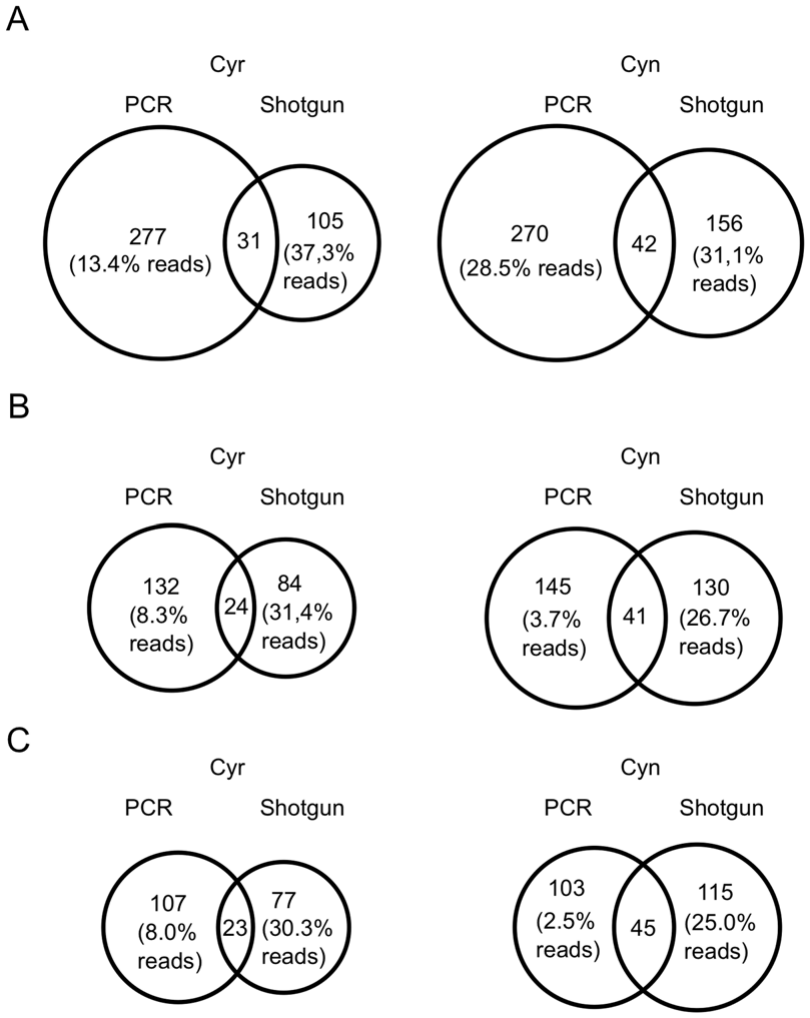
Shared and unique OTUs of the PCR-based and shotgun-based sponge datasets. Circle sizes are proportional to OTU number. (A) 0.01 phylogenetic distance OTU. (B) 0.03 phylogenetic distance OTU. (C) 0.05 phylogenetic distance OTU.

We then compared the pyro-tag-sequencing data and the 16S rRNA gene reconstruction approach by generating OTUs at different phylogenetic distance cut-offs (Materials and Methods). In general, the PCR-based approach produced more OTUs than the metagenome-based approach, except at the 0.05 OTU-level for *C. concentrica* (Figure 4). This is obviously because of the much higher sequencing depth for the 16S rRNA gene in the pyro-tag samples (Table 2, 3). A relative low number of common OTUs were observed between the two approaches. However, the OTUs unique to the PCR-based approach only present a low proportion (2.5–8.3%) of all pyro-tag reads at OTU-levels of 0.03 and 0.05. This result shows that the majority of pyro-tag reads come from phylotypes that are also contained in the metagenomic data set and that the unique OTUs of the PCR-based approach either constitute low abundance phylotypes (e.g. are part of the rare biosphere) [43] or are undetected chimeras [44]. In contrast, a high proportion of reads (∼30%) belong to unique OTUs generated from the 16S rRNA gene reconstruction, which indicates that they come from abundant organisms that were missed by PCR-based approaches. Different levels of diversity of the PCR analysis and metagenomic reconstruction are also reflected in rarefaction plots (Figure S1). Although the sampling depths of the shotgun samples were relatively low, the trends reflected in their rarefaction plots compared to the plots of the PCR samples clearly suggests a higher community diversity.

### Primer bias can explain the lack of OTU detection

**Figure 5:**
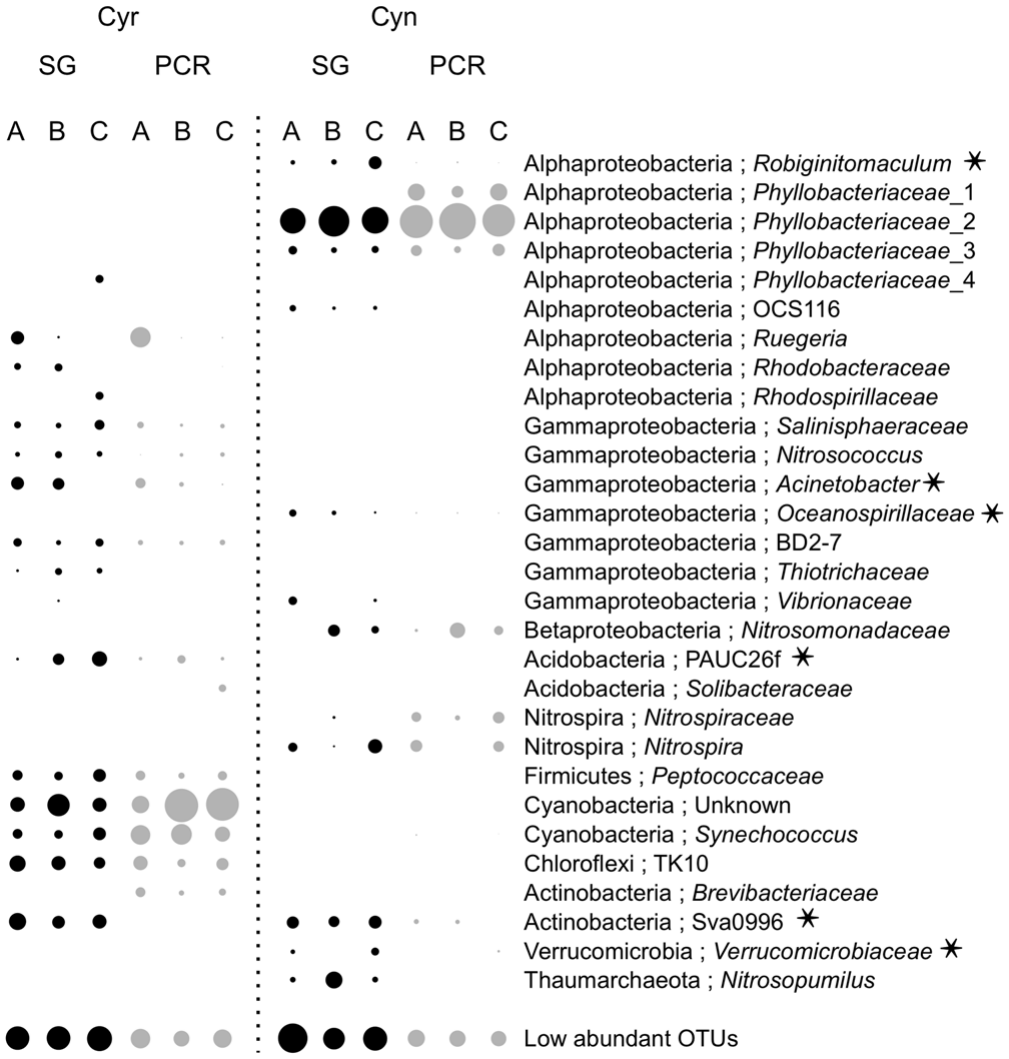
Abundance and primer-mismatches in the top OTUs at the 0.01 phylogenetic distance level for the sponge datasets. Asterisk, primer-mis-match event.

To further investigate how PCR-amplification failed to detect certain groups of bacteria (see above), we taxonomically classified the most abundant 0.01-level OTUs (>2% in any of the 12 samples) (Figure 5). OTUs assigned to the bacterial groups of *Robiginitomaculum*, *Phyllobacteriaceae*_4, OCS116, *Rhodobacteraceae*, *Rhodospirillaceae*, *Acinetobacter*, *Oceanospirillaceae, Thiotrichaceae, Vibrionaceae*, PAUC26f, Sva0996 and *Verrucomicrobiaceae* were consistently missed or poorly recovered by PCR. Among them, eight 16S rRNA gene contigs belonging to seven 0.01 OTUs (i.e. *Robiginitomaculum*, *Rhodobacteraceae*, *Acinetobacter*, *Oceanospirillaceae*, PAUC26f, Sva0996, and *Verrucomicrobiaceae*, including two contigs belonging to Sva0996) covered the entire V1-3 region of the 16S rRNA gene. Alignment of these eight contigs to the degenerate primers 28F/519R found seven of them had mismatches (either one or both primers) (asterisks in Figure 5). This suggests that primer bias is one of the major causes for the PCR-based approach missing certain OTUs (Figure 4).

### Phylogenetic analysis of the novel 16S rRNA sequences detected by the shotgunapproach

To examine how many of the 25 16S rRNA gene contigs reconstructed from shotgun sequencing data have so far not been detected by PCR-based approaches in these two sponges, we performed searches against the NCBI nt database (7 April 2012) and the full-length 16S rRNA genes (primes 27F and 1492R) previously amplified from *C. concentrica* by Thomas *et al.* [45]. Any match with a BlastN identity of >99% was considered as an amplicon counterpart to the contigs. While none of the 13 contigs from *C. coralliophila* found amplicon counterparts, 10 of the 12 contigs from *C. concentrica* had been previously detected (Table S2).

Among the 15 undetected sequences, ten were amplified by the primers used in the present study (Figure 5). Of the five remaining contigs, the archaeon*Nitrosopumilus* has been previously detected from the functional metaproteogenomic study of *C. concentrica* [46]. The four bacterial contigs were classified as Sva0996, *Rhodobacteraceae*, BD2-11 and *Oceanospirillaceae* (Table S2) and then further phylogenetically analyzed (Figure S2). The Acidimicrobiales- and the Gemmatimonadetes-phylotypes are part of sponge/coral specific clades in the Sva0996 group and the BD2-11 group, respectively (Figure S2B, C). The *Rhodobacteraceae*-phylotype branches distantly from the most closely related free-living neighbors (Figure S2A). The *Oceanospirillaceae*-phylotype has a closely related free-living strain (Figure S2D). This phylotype in the sponge *C. concentrica* has been consistently missed by PCR-based approaches despite current and previous extensive sequencing efforts using different protocols and primers [45], [47]–[49].

## Discussion

In the present study, we describe how stringent assemblies and filtering can recover nearly full-length 16S rRNA gene sequences from metagenomicpyrosequencing datasets. Through simulation of communities with various complexities, we show that chimera formation is minimal and will not impact on prediction of community composition. These properties make the described approach readily applicable to existing and future metagenomic datasets. Advances in next generation sequencing technology have in recent years led to a surge of metagenomic studies and thousands of datasets are currently available [50], [51]. Our approach will thus prove itself useful in defining the phylogenetic diversity and community composition harbored in these metagenomic resources. We are also expecting that this will lead to the discovery of new phylotypes that have previously eluded PCR-based detection and our analysis of sponge symbiont communities has provided examples of this.

Pyro-tag-sequencing has been become a standard approach for defining community composition and has thus been extensively applied in, for example, the Human Microbiome Project [52] and clinical diagnosis [53]. We show here that PCR can cause a substantial impact on the assessment of communities in terms of diversity, composition and abundance. It might therefore be worthwhile to benchmark primer choice based on 16S rRNA genes reconstructed from metagenomic data before establishing routine assays based on PCR methods.

## Supporting Information

Figure S1 **Rarefaction plots for the sponge datasets.**Dataare based on an OTU distance of 0.01 (A), 0.03 (B), and 0.05 (C), and based on phylogenetic distance (D). The plots on the right are enlargements of the dashed boxes on the diagrams to the left.
(TIFF)

Figure S2 **Phylogenetic analysis of the 16S rRNA gene sequences missed by PCR.**Percentage bootstrapping values (1,000 replications) greater than 50% are shown. Sponge-derived sequences are shown in bold. Pentagram-marked sequences are from the present study. (A) The *Rhodobacteraceae* bacterium in the family *Rhodobacteraceae*, with tree rooted to *Leisingeramethylohalidivoraans* [AY005463]. (B) the*Acidimicrobiales* bacterium in the clade Sva0996, with tree rooted to *Iamiamajanohamensis* [AB360448]. (C) The *Gemmatimonadetes* (class) bacterium in the clade BD2-11, with tree rooted to *Gemmatimonasaurantiaca* [AP009153]. (D) The *Oceanospirillaceae* bacterium in the family *Oceanospirillaceae*, with tree rooted to *Comamonascomposti* [EF015884].
(PNG)

Table S1 **Simulated datasets.**
(DOCX)

Table S2 **16S rRNA gene contigs generated from sponge metagenomic samples.**
(DOCX)
