# Supplementary figures and images for: Reconstruction of Ribosomal RNA Genes from Metagenomic Data
Source: PLoS One. 2012 Jun 27;7(6):e39948. doi: 10.1371/journal.pone.0039948 (PMC3384625; doi:10.1371/journal.pone.0039948)

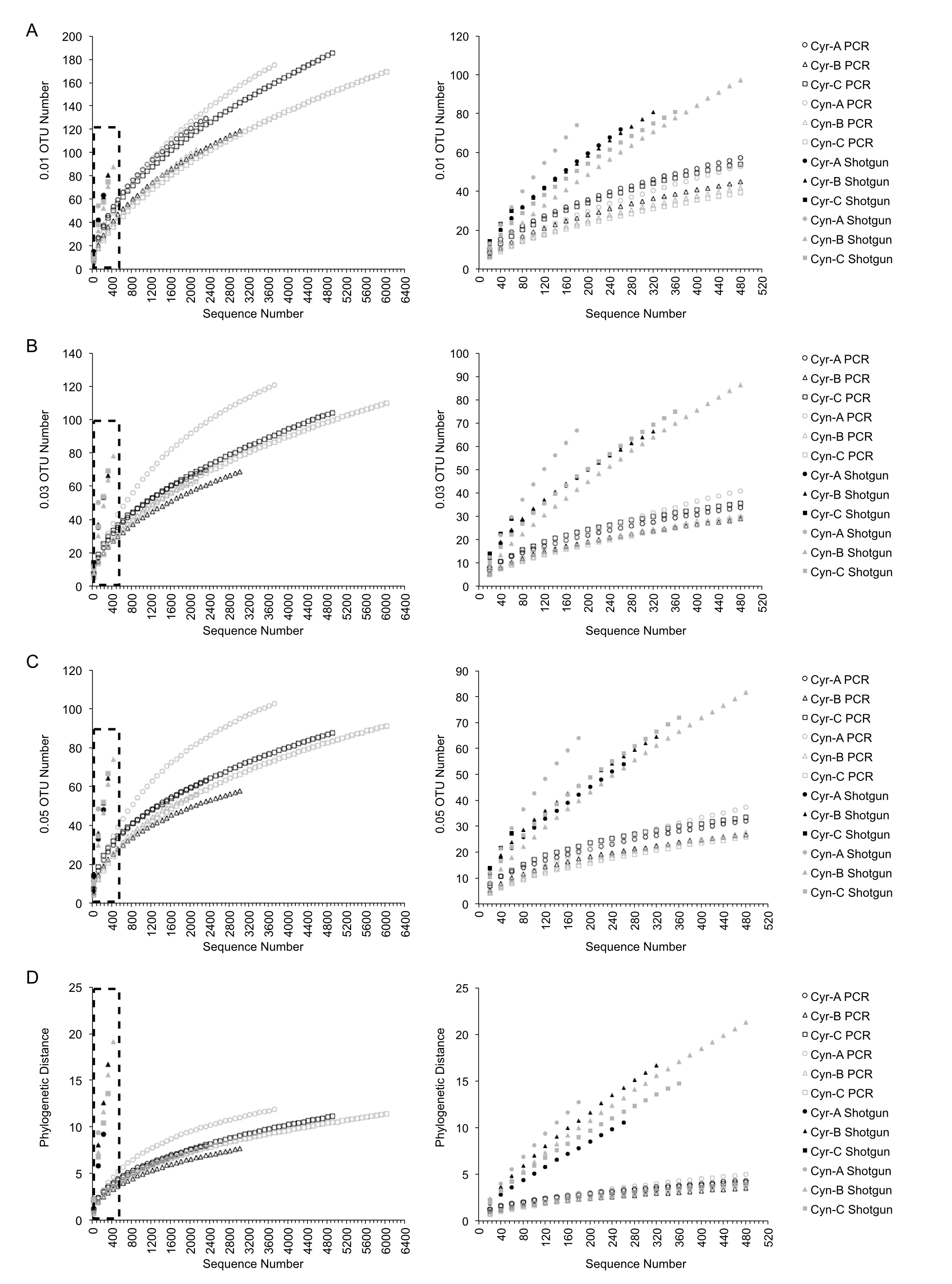

Supplement: Figure S1 — Rarefaction plots for the sponge datasets.Dataare based on an OTU distance of 0.01 (A), 0.03 (B), and 0.05 (C), and based on phylogenetic distance (D). The plots on the right are enlargements of the dashed boxes on the diagrams to the left. (TIFF) [file pone.0039948.s001.tiff]

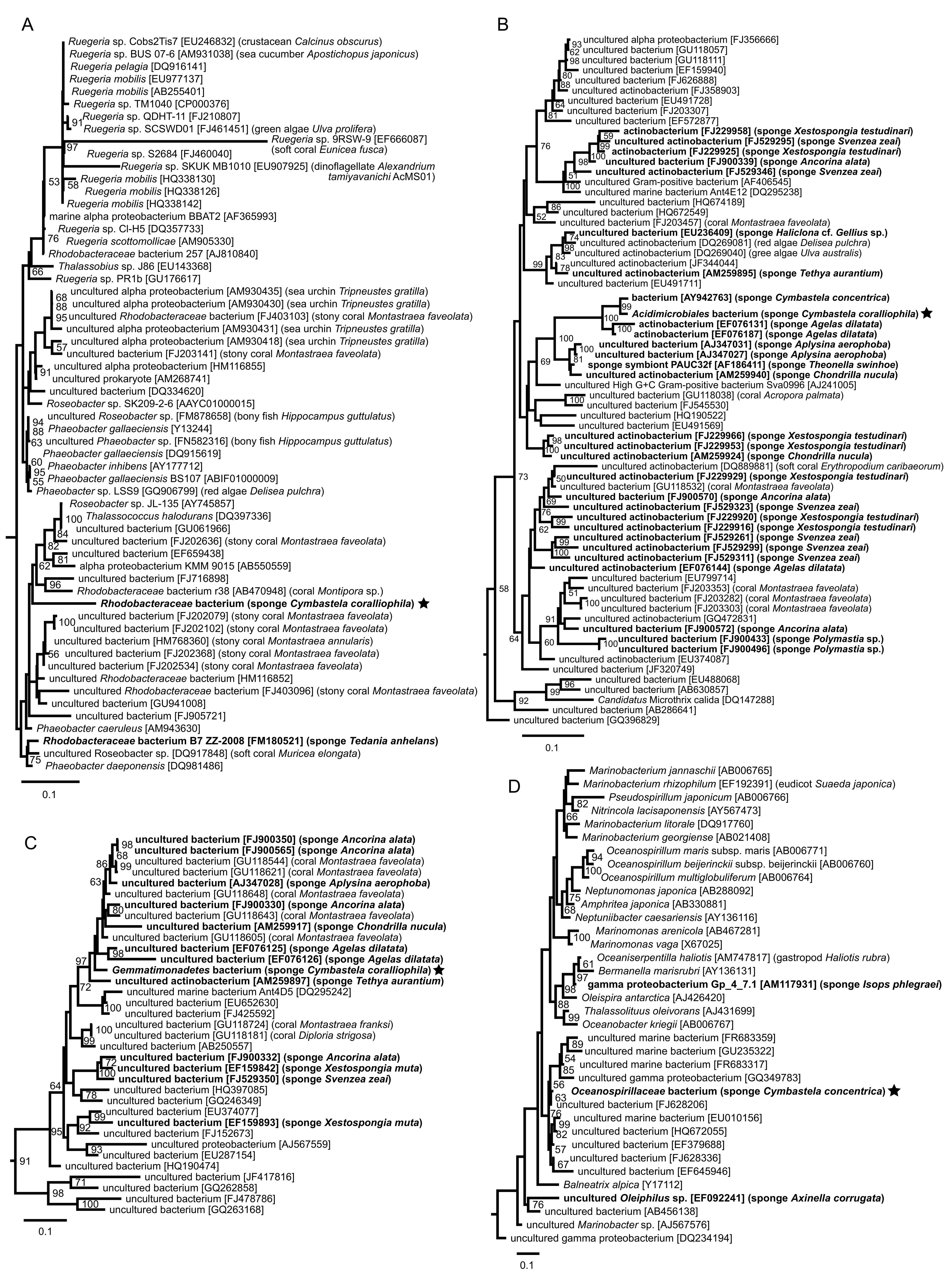

Supplement: Figure S2 — Phylogenetic analysis of the 16S rRNA gene sequences missed by PCR.Percentage bootstrapping values (1,000 replications) greater than 50% are shown. Sponge-derived sequences are shown in bold. Pentagram-marked sequences are from the present study. (A) The Rhodobacteraceae bacterium in the family Rhodobacteraceae, with tree rooted to Leisingeramethylohalidivoraans [AY005463]. (B) theAcidimicrobiales bacterium in the clade Sva0996, with tree rooted to Iamiamajanohamensis [AB360448]. (C) The Gemmatimonadetes (class) bacterium in the clade BD2-11, with tree rooted to Gemmatimonasaurantiaca [AP009153]. (D) The Oceanospirillaceae bacterium in the family Oceanospirillaceae, with tree rooted to Comamonascomposti [EF015884]. (PNG) [file pone.0039948.s002.png]
