# Supplementary material for: Reconstruction of Ribosomal RNA Genes from Metagenomic Data
Source: PLoS One. 2012 Jun 27;7(6):e39948. doi: 10.1371/journal.pone.0039948 (PMC3384625; doi:10.1371/journal.pone.0039948)
Supplement: Table S1 — Simulated datasets. (DOCX) [file pone.0039948.s003.docx]

**Table S1.** Simulated datasets.

| **Domain** | **Organism** | **IMG Taxon Object ID** | **NCBI Taxon ID** | **Genome size** | **16S genes** | **Unique 16S genes** | **HC-A reads** | **HC-B reads** | **HC-C reads** | **MC-A reads** | **MC-B reads** | **MC-C reads** | **LC-A reads** | **LC-B reads** | **LC-C reads** |
| --- | --- | --- | --- | --- | --- | --- | --- | --- | --- | --- | --- | --- | --- | --- | --- |
| Archaea | *Archaeoglobus fulgidus* VC-16, DSM 4304 | 638154502 | 224325 | 2178400 | 1 | 1 | 9061 | 8584 | 13352 | 7940 | 5138 | 5138 | 21752 | 6647 | 7335 |
| Archaea | Candidatus *Korarchaeum cryptofilum* OPF8 | 641522611 | 374847 | 1590757 | 1 | 1 | 19075 | 9537 | 11922 | 6539 | 6539 | 7006 | 7251 | 6647 | 6724 |
| Archaea | *Desulfurococcus mucosus* DSM 2162 | 649633040 | 765177 | 1314639 | 1 | 1 | 8584 | 9537 | 9061 | 6539 | 5138 | 6072 | 6647 | 6647 | 5501 |
| Archaea | *Halobacterium salinarum* R1, DSM 671 | 641522631 | 478009 | 2668776 | 1 | 1 | 7153 | 8107 | 8584 | 5138 | 5605 | 3737 | 6042 | 5438 | 4890 |
| Archaea | *Ignicoccus hospitalis* KIN4/I, DSM 18386 | 640753029 | 453591 | 1297538 | 1 | 1 | 8107 | 11445 | 19075 | 4671 | 5605 | 4671 | 6042 | 6647 | 6112 |
| Archaea | *Methanocaldococcus jannaschii* DSM 2661 | 638154505 | 243232 | 1739916 | 2 | 1 | 8584 | 9061 | 11922 | 5605 | 5138 | 4671 | 5438 | 7251 | 4890 |
| Archaea | *Methanococcus voltae* A3 | 646564549 | 456320 | 1936387 | 2 | 1 | 11445 | 10014 | 8107 | 5605 | 6072 | 4671 | 5438 | 4834 | 6112 |
| Archaea | *Methanosarcina barkeri* Fusaro, DSM 804 | 637000162 | 269797 | 4873766 | 3 | 1 | 8584 | 9537 | 11445 | 5138 | 7473 | 5605 | 5438 | 7251 | 6724 |
| Archaea | *Methanospirillum hungatei* JF-1 | 637000164 | 323259 | 3544738 | 4 | 1 | 11445 | 8584 | 8584 | 5605 | 5138 | 5605 | 6647 | 4230 | 5501 |
| Archaea | *Pyrobaculum aerophilum* IM2 | 638154513 | 178306 | 2222430 | 1 | 1 | 11445 | 11445 | 9537 | 140121 | 129379 | 162074 | 5438 | 4230 | 7335 |
| Archaea | *Pyrobaculum islandicum* DSM 4184 | 639633053 | 384616 | 1826402 | 1 | 1 | 10491 | 10968 | 10014 | 5138 | 13078 | 13078 | 4834 | 6647 | 7335 |
| Archaea | *Staphylothermus marinus* F1, DSM 3639 | 640069332 | 399550 | 1570485 | 1 | 1 | 10491 | 10014 | 10014 | 5138 | 5138 | 4204 | 4834 | 7251 | 5501 |
| Archaea | *Sulfolobus islandicus* Y.N.15.51 | 643692049 | 419942 | 2854410 | 1 | 1 | 10968 | 11445 | 11922 | 4204 | 5138 | 5138 | 5438 | 5438 | 8557 |
| Archaea | *Thermococcus gammatolerans* EJ3 | 644736411 | 593117 | 2045438 | 1 | 1 | 10014 | 11445 | 11445 | 3737 | 4671 | 4204 | 4230 | 5438 | 6112 |
| Bacteria | *Acidobacterium* sp. MP5ACTX9 | 649633002 | 696844 | 5503984 | 1 | 1 | 10014 | 11445 | 10491 | 11210 | 5138 | 4671 | 7251 | 6647 | 6724 |
| Bacteria | *Acinetobacter baumannii* ATCC 17978 | 640069301 | 400667 | 4001457 | 5 | 1 | 8107 | 9061 | 11445 | 5605 | 5605 | 5138 | 6647 | 6042 | 6724 |
| Bacteria | *Actinobacillus succinogenes* 130Z | 640753001 | 339671 | 2046146 | 6 | 1 | 11445 | 10968 | 9537 | 6539 | 4204 | 4671 | 7251 | 8459 | 7335 |
| Bacteria | *Agrobacterium vitis* S4 | 643348505 | 311402 | 6320946 | 4 | 1 | 8584 | 11922 | 11445 | 5138 | 5138 | 4671 | 6647 | 4834 | 4279 |
| Bacteria | *Alkalilimnicola ehrlichei* MLHE-1 | 637000005 | 187272 | 3272789 | 2 | 1 | 11922 | 11922 | 10491 | 5138 | 5605 | 4204 | 6647 | 4230 | 6724 |
| Bacteria | *Anabaena variabilis* ATCC 29413 | 646564504 | 240292 | 7105752 | 4 | 1 | 10491 | 11445 | 9537 | 5138 | 5138 | 8407 | 6647 | 6647 | 7335 |
| Bacteria | *Anaeromyxobacter dehalogenans* 2CP-C | 637000007 | 290397 | 5013479 | 2 | 1 | 11922 | 11445 | 10968 | 5138 | 3737 | 5138 | 6647 | 8459 | 6724 |
| Bacteria | *Arthrobacter* sp. FB24 | 639633006 | 290399 | 5011599 | 5 | 1 | 10968 | 10968 | 8107 | 5138 | 4204 | 5138 | 6647 | 6042 | 5501 |
| Bacteria | *Bacillus pseudofirmus* OF4 | 646311908 | 398511 | 4249248 | 7 | 1 | 11445 | 11922 | 10491 | 6539 | 4671 | 5138 | 7251 | 7251 | 7335 |
| Bacteria | *Bartonella quintana* Toulouse | 637000028 | 283165 | 1581384 | 2 | 1 | 8107 | 11445 | 11922 | 7473 | 5138 | 6072 | 7251 | 6647 | 6724 |
| Bacteria | *Bifidobacterium longum* DJO10A | 642555107 | 205913 | 2375286 | 4 | 1 | 10014 | 11922 | 11445 | 3737 | 5605 | 5605 | 6042 | 6647 | 8557 |
| Bacteria | *Bradyrhizobium* sp. BTAi1 | 640427103 | 288000 | 8422430 | 2 | 1 | 11922 | 10968 | 12399 | 129379 | 47641 | 47641 | 67069 | 56193 | 128973 |
| Bacteria | *Burkholderia cenocepacia* AU 1054 | 637000046 | 331271 | 7249477 | 6 | 1 | 10968 | 11445 | 12399 | 5138 | 4671 | 4671 | 6647 | 6647 | 6724 |
| Bacteria | *Burkholderia cenocepacia* HI2424 | 639633014 | 331272 | 8139086 | 6 | 1 | 10968 | 11922 | 11445 | 5138 | 5138 | 6539 | 6647 | 7251 | 3667 |
| Bacteria | *Burkholderia xenovorans* LB400 | 637000053 | 266265 | 9731138 | 6 | 1 | 9061 | 9061 | 9537 | 4204 | 4671 | 5138 | 5438 | 6647 | 5501 |
| Bacteria | *Campylobacter concisus* 13826 | 640753009 | 360104 | 2099412 | 3 | 1 | 11445 | 11445 | 10491 | 6539 | 4671 | 3737 | 7251 | 4230 | 3667 |
| Bacteria | Candidatus *Riesia pediculicola* USDA | 646564517 | 515618 | 582127 | 2 | 1 | 9537 | 10014 | 11922 | 6539 | 5605 | 5138 | 6647 | 6647 | 6724 |
| Bacteria | *Cellulophaga lytica* LIM-21, DSM 7489 | 649633032 | 867900 | 3765936 | 4 | 1 | 9061 | 10968 | 7153 | 4671 | 5138 | 4671 | 6647 | 6042 | 4279 |
| Bacteria | *Chloroflexus aurantiacus* J-10-fl | 641228485 | 324602 | 5193782 | 3 | 2 | 11445 | 8584 | 10968 | 5605 | 5138 | 5138 | 7855 | 7855 | 22005 |
| Bacteria | *Clostridium beijerinckii* NCIMB 8052 | 640753016 | 290402 | 6000632 | 14 | 1 | 9537 | 11922 | 10014 | 6072 | 6072 | 5138 | 3625 | 6042 | 6724 |
| Bacteria | *Clostridium perfringens* 13 | 637000079 | 195102 | 3085740 | 10 | 1 | 9061 | 10968 | 11445 | 8407 | 5138 | 6539 | 3021 | 6042 | 4279 |
| Bacteria | *Clostridium thermocellum* ATCC 27405 | 640069309 | 203119 | 3894953 | 4 | 1 | 10491 | 11445 | 10968 | 5605 | 4671 | 5138 | 6647 | 6042 | 5501 |
| Bacteria | *Cytophaga hutchinsonii* ATCC 33406 | 637000087 | 269798 | 4433218 | 3 | 1 | 9537 | 10014 | 8584 | 4671 | 5138 | 4204 | 56193 | 67069 | 56846 |
| Bacteria | *Dechloromonas aromatica* RCB | 637000088 | 159087 | 4501104 | 4 | 1 | 10014 | 11922 | 11445 | 4671 | 3737 | 5138 | 6042 | 7251 | 7335 |
| Bacteria | *Desulfitobacterium hafniense* DCB-2 | 643348537 | 272564 | 6083768 | 5 | 3 | 10014 | 11922 | 11445 | 4671 | 5605 | 5605 | 6647 | 5438 | 4890 |
| Bacteria | *Desulfovibrio desulfuricans* G20 | 637000095 | 207559 | 3730232 | 4 | 1 | 10491 | 9537 | 25274 | 4671 | 5605 | 8407 | 6647 | 6647 | 7335 |
| Bacteria | *Dickeya zeae* Ech1591 | 644736355 | 561229 | 4813854 | 7 | 1 | 11445 | 25274 | 10014 | 8407 | 4671 | 4671 | 6647 | 7251 | 6112 |
| Bacteria | *Ehrlichia canis* Jake | 637000097 | 269484 | 1315030 | 1 | 1 | 10014 | 10968 | 10014 | 6539 | 5138 | 5138 | 8459 | 4834 | 6112 |
| Bacteria | *Escherichia coli* O6:K15:H31 536 (UPEC) | 637000104 | 362663 | 4938920 | 7 | 2 | 9061 | 12399 | 10014 | 5138 | 4671 | 4671 | 6042 | 6042 | 8557 |
| Bacteria | *Frankia* sp. CcI3 | 637000116 | 106370 | 5433628 | 2 | 1 | 10968 | 11922 | 10968 | 5138 | 5605 | 5605 | 6647 | 6647 | 6112 |
| Bacteria | *Frankia* sp. EAN1pec | 641228492 | 298653 | 9081415 | 3 | 1 | 10968 | 11445 | 9537 | 5138 | 5138 | 5138 | 6647 | 6042 | 7335 |
| Bacteria | *Geobacter metallireducens* GS-15 | 637000119 | 269799 | 4011182 | 3 | 3 | 10491 | 9061 | 9061 | 5138 | 5138 | 5138 | 6647 | 3625 | 6724 |
| Bacteria | *Halothiobacillus neapolitanus* c2 | 646311935 | 555778 | 2582886 | 2 | 1 | 12399 | 13352 | 10968 | 4671 | 7006 | 11210 | 6042 | 6042 | 7335 |
| Bacteria | *Helicobacter pylori* J99 | 637000134 | 85963 | 1643831 | 2 | 1 | 12399 | 11445 | 11922 | 4671 | 6072 | 5605 | 6042 | 6042 | 5501 |
| Bacteria | *Jannaschia* sp. CCS1 | 637000137 | 290400 | 4404049 | 1 | 1 | 11922 | 8584 | 10491 | 5605 | 5138 | 5605 | 7251 | 7251 | 6724 |
| Bacteria | *Kineococcus radiotolerans* SRS30216 | 640753031 | 266940 | 4893957 | 4 | 1 | 11922 | 10491 | 11445 | 5605 | 5138 | 5138 | 7251 | 7251 | 6724 |
| Bacteria | *Kribbella flavida* DSM 17836 | 646311938 | 479435 | 7579488 | 2 | 1 | 10968 | 11922 | 10491 | 4671 | 4204 | 5138 | 6042 | 6042 | 5501 |
| Bacteria | *Magnetococcus* sp. MC-1 | 639633036 | 156889 | 4628740 | 3 | 1 | 10968 | 11445 | 11922 | 4671 | 4671 | 6539 | 6042 | 6647 | 6724 |
| Bacteria | *Marinobacter aquaeolei* VT8 | 639633037 | 351348 | 4647952 | 3 | 1 | 11445 | 12399 | 11445 | 5605 | 5138 | 5138 | 6647 | 6647 | 6724 |
| Bacteria | *Moorella thermoacetica* ATCC 39073 | 637000167 | 264732 | 2628784 | 1 | 1 | 25274 | 12399 | 11445 | 13078 | 4671 | 5605 | 15106 | 6647 | 3056 |
| Bacteria | *Mycobacterium ulcerans* Agy99 | 642555140 | 362242 | 5805761 | 1 | 1 | 10968 | 10968 | 9061 | 5138 | 5605 | 6539 | 5438 | 5438 | 6724 |
| Bacteria | *Nitrobacter winogradskyi* Nb-255 | 637000193 | 323098 | 3402093 | 1 | 1 | 11445 | 11922 | 23367 | 4671 | 4204 | 5605 | 7251 | 7855 | 4890 |
| Bacteria | *Nitrosospira multiformis* ATCC 25196 | 637000197 | 323848 | 3234309 | 1 | 1 | 11445 | 8107 | 11922 | 5605 | 5605 | 7940 | 6647 | 15106 | 7335 |
| Bacteria | *Nocardioides* sp. JS614 | 639633046 | 196162 | 5394058 | 2 | 1 | 11445 | 10968 | 11922 | 5605 | 5138 | 5138 | 6647 | 5438 | 6724 |
| Bacteria | *Oenococcus oeni* PSU-1 | 639633047 | 203123 | 1782786 | 2 | 1 | 9537 | 9537 | 10968 | 4671 | 4671 | 5605 | 4834 | 3021 | 7335 |
| Bacteria | *Paracoccus denitrificans* PD1222 | 639633048 | 318586 | 5175736 | 3 | 1 | 11922 | 10014 | 11445 | 5138 | 7006 | 4671 | 6042 | 5438 | 4279 |
| Bacteria | *Pelobacter carbinolicus* DSM 2380 | 637000204 | 338963 | 3662252 | 2 | 1 | 10014 | 7153 | 10968 | 5138 | 6539 | 5138 | 6647 | 5438 | 6112 |
| Bacteria | *Petrotoga mobilis* SJ95 | 641228500 | 403833 | 2169548 | 2 | 1 | 10014 | 11445 | 10968 | 5138 | 3737 | 5138 | 5438 | 6647 | 6112 |
| Bacteria | *Polaromonas* sp. JS666 | 637000208 | 296591 | 5898676 | 1 | 1 | 11445 | 10014 | 11922 | 6072 | 5605 | 5605 | 7251 | 4230 | 6724 |
| Bacteria | *Pseudoalteromonas atlantica* T6c | 637000216 | 342610 | 5094958 | 5 | 1 | 12399 | 8584 | 12399 | 6072 | 4204 | 5138 | 7251 | 6647 | 6724 |
| Bacteria | *Pseudomonas putida* F1 | 640427132 | 351746 | 5925059 | 6 | 1 | 12399 | 12399 | 8107 | 5605 | 6539 | 6072 | 6647 | 6647 | 4890 |
| Bacteria | *Rhizobium leguminosarum* bv. trifolii WSM1325 | 644736401 | 395491 | 7418122 | 3 | 1 | 10014 | 23367 | 10968 | 4204 | 5605 | 5138 | 4834 | 5438 | 6112 |
| Bacteria | *Rhodobacter sphaeroides* 2.4.1 | 640069327 | 272943 | 4603060 | 3 | 1 | 8584 | 19075 | 10968 | 3737 | 3737 | 5138 | 4834 | 8459 | 6724 |
| Bacteria | *Rhodococcus equi* 103S | 649633089 | 685727 | 5043170 | 4 | 2 | 11922 | 10968 | 9061 | 5138 | 5138 | 5138 | 4834 | 6647 | 6112 |
| Bacteria | *Rhodopseudomonas palustris* BisA53 | 639279312 | 316055 | 5502424 | 2 | 1 | 11922 | 11922 | 10014 | 5605 | 6539 | 5138 | 6647 | 6647 | 4279 |
| Bacteria | *Rhodopseudomonas palustris* BisB18 | 637000237 | 316056 | 5513844 | 2 | 1 | 11922 | 12399 | 11922 | 52779 | 52779 | 52779 | 7855 | 6042 | 6724 |
| Bacteria | *Rhodopseudomonas palustris* BisB5 | 637000238 | 316057 | 4892717 | 2 | 1 | 11922 | 11445 | 11445 | 162074 | 140121 | 129379 | 7251 | 7251 | 6112 |
| Bacteria | *Rhodopseudomonas palustris* HaA2 | 637000240 | 316058 | 5331656 | 1 | 1 | 11445 | 9061 | 11445 | 5605 | 11210 | 6539 | 313595 | 313595 | 244499 |
| Bacteria | *Rhodospirillum rubrum* ATCC 11170 | 637000241 | 269796 | 4406557 | 4 | 1 | 10491 | 10491 | 10968 | 47641 | 162074 | 140121 | 6647 | 7251 | 6724 |
| Bacteria | *Rubrobacter xylanophilus* DSM 9941 | 637000248 | 266117 | 3299423 | 1 | 1 | 23367 | 10014 | 12399 | 7006 | 5605 | 5605 | 8459 | 3625 | 6724 |
| Bacteria | *Saccharophagus degradans* 2-40 | 637000249 | 203122 | 5057531 | 2 | 1 | 11445 | 10491 | 11922 | 5138 | 3737 | 5138 | 6042 | 5438 | 7335 |
| Bacteria | *Shewanella baltica* OS155 | 640069330 | 325240 | 5084318 | 10 | 2 | 11445 | 10014 | 10491 | 5605 | 8407 | 6539 | 3625 | 4834 | 4890 |
| Bacteria | *Shewanella* sp. ANA-3 | 639633058 | 94122 | 5100729 | 9 | 1 | 11922 | 10491 | 10491 | 5138 | 5138 | 4671 | 6042 | 6042 | 7946 |
| Bacteria | *Shewanella* sp. MR-7 | 637000260 | 60481 | 4546355 | 9 | 2 | 12399 | 10491 | 10014 | 5138 | 4671 | 6539 | 4230 | 6647 | 7946 |
| Bacteria | *Shewanella* sp. W3-18-1 | 639633059 | 351745 | 4754010 | 8 | 1 | 11922 | 10014 | 8584 | 5138 | 5138 | 5138 | 6042 | 6647 | 15281 |
| Bacteria | *Sphingopyxis alaskensis* RB2256 | 637000271 | 317655 | 3343420 | 1 | 1 | 11445 | 10491 | 11445 | 5138 | 8407 | 4671 | 7251 | 21752 | 6724 |
| Bacteria | *Streptococcus pyogenes* M28, MGAS6180 | 637000298 | 319701 | 1897573 | 6 | 1 | 12399 | 10491 | 11445 | 5138 | 5605 | 5605 | 5438 | 7251 | 5501 |
| Bacteria | *Streptococcus thermophilus* LMD-9 | 639633062 | 322159 | 1842121 | 6 | 2 | 10968 | 10491 | 8584 | 3737 | 4671 | 5605 | 4230 | 6647 | 4890 |
| Bacteria | *Syntrophobacter fumaroxidans* MPOB | 639633063 | 335543 | 4848841 | 2 | 1 | 10491 | 10014 | 9061 | 4671 | 6539 | 3737 | 6647 | 6647 | 6112 |
| Bacteria | *Thermobifida fusca* YX | 637000319 | 269800 | 3642249 | 4 | 1 | 9537 | 11922 | 10014 | 4204 | 5138 | 5605 | 5438 | 6042 | 6112 |
| Bacteria | *Thermotoga neapolitana* DSM 4359 | 643348584 | 309803 | 1884562 | 1 | 1 | 11445 | 11445 | 11445 | 5138 | 6539 | 5138 | 4230 | 4834 | 7335 |
| Bacteria | *Thiobacillus denitrificans* ATCC 25259 | 637000324 | 292415 | 2909809 | 2 | 1 | 13352 | 12399 | 12399 | 7006 | 7940 | 3737 | 8459 | 7251 | 6724 |
| Bacteria | *Thiomicrospira crunogena* XCL-2 | 637000325 | 317025 | 2427734 | 3 | 1 | 10968 | 11445 | 11922 | 5138 | 5138 | 7473 | 6042 | 6647 | 6724 |
| Bacteria | *Trichodesmium erythraeum* IMS101 | 637000329 | 203124 | 7750108 | 2 | 1 | 11922 | 8107 | 10014 | 5138 | 5138 | 4204 | 7251 | 7251 | 6112 |
| Bacteria | *Waddlia chondrophila* WSU 86-1044 | 646564588 | 716544 | 2131905 | 2 | 1 | 11445 | 10968 | 11445 | 4204 | 6539 | 7006 | 4230 | 4834 | 6724 |
| Bacteria | *Xylella fastidiosa* M12 | 641522659 | 405440 | 2475130 | 2 | 1 | 10014 | 11445 | 12399 | 3737 | 5138 | 3737 | 4834 | 4834 | 5501 |
