# Supplementary material for: Reconstruction of Ribosomal RNA Genes from Metagenomic Data
Source: PLoS One. 2012 Jun 27;7(6):e39948. doi: 10.1371/journal.pone.0039948 (PMC3384625; doi:10.1371/journal.pone.0039948)
Supplement: Table S2 — 16S rRNA gene contigs generated from sponge metagenomic samples. (DOCX) [file pone.0039948.s004.docx]

**Table S2.** 16S rRNA gene contigs generated from sponge metagenomic samples.

| **16S rRNA contig** | **Length (nt)** | **Classification** | **Amplicon counterpart** |
| --- | --- | --- | --- |
| **Cyr** |  |  |  |
| contig00001 | 1465 | Cyanobacteria | this study |
| contig00002 | 1509 | Sva0996 | - |
| contig00003 | 1481 | TK10 | this study |
| contig00004 | 1518 | Acinetobacter | this study |
| contig00005 | 1445 | *Rhodobacteraceae* | - |
| contig00006 | 1512 | BD2-7 | this study |
| contig00007 | 1535 | *Peptococcaceae* | this study |
| contig00008 | 1473 | PAUC26f | this study |
| contig00009 | 1442 | Ruegeria | this study |
| contig00010 | 1315 | *Synechococcus* | this study |
| contig00011 | 1232 | *Salinisphaeraceae* | this study |
| contig00012 | 1218 | *Nitrosococcus* | this study |
| contig00013 | 1283 | BD2-11 | - |
| **Cyn** |  |  |  |
| contig00001 | 1508 | Sva0996 | AY942763, ref. (45), this study |
| contig00002 | 1459 | *Robiginitomaculum* | AY942765, ref. (45) |
| contig00003 | 1517 | *Nitrosomonadaceae* | ref. (45), this study |
| contig00004 | 1458 | *Nitrosopumilus* | - |
| contig00005 | 1516 | *Nitrospira* | AY942775, AY942757, ref. (45), this study |
| contig00006 | 1466 | *Phyllobacteriaceae*_2 | AY942778, AY942764, ref. (45), this study |
| contig00008 | 1383 | *Oceanospirillaceae* | - |
| contig00009 | 1436 | *Aurantimonadaceae* | ref. (45), this study |
| contig00011 | 777 | *Verrucomicrobiaceae* | AY942771, AY942760, ref. (45) |
| contig00012 | 1172 | OCS116 | AY942776, GQ160462, ref. (45) |
| contig00021 | 493 | *Mesorhizobium* | ref. (45) |
| contig00022 | 494 | *Phyllobacteriaceae*_3 | ref. (45), this study |
